# High Grade B- Cell Non- Hodgkin’s Lymphoma Arising in a Mature Cystic Teratoma of The Ovary: A Case Report

**DOI:** 10.22088/BUMS.6.4.239

**Published:** 2018-02-10

**Authors:** Sameen Afzal, Samina Zaman

**Affiliations:** *Department of Histopathology, Chughtai Lab Lahore, Lahore, Pakistan.*

**Keywords:** Lymphoma, teratoma, ovary, germ cell neoplasms

## Abstract

Mature cystic teratoma (MCT) is the most common type of ovarian germ cell tumor occurring in females of reproductive age. It is typically benign, but rare malignant transformations have been reported in 1-2% of the cases. Among a wide variety of malignancies arising in MCTs, high grade lymphomas are the least common. We present a case of a 45- years -old female with a unilateral adnexal mass. Gross examination revealed a unilocular cyst with a smooth and intact capsule. The cyst lumen was filled with sebaceous material and hair. Except for a 5.0 cm Rokitansky nodule, no other nodule or papillary structures were identified. Microscopic examination revealed an array of mature tissues arising from different germ cell layers, and foci of diffuse sheets of large atypical lymphoid cells. These were positive for CD-20 marker, confirming their B lymphoid series cell origin. A final diagnosis of a high grade B cell non Hodgkin’s lymphoma arising in an ovarian MCT was made. Such cases have been known to be associated with a very poor prognosis, and there are no established criteria for their pre-operative diagnosis. Risk factors for malignant transformation in an MCT including tumor size, post menopausal status and serum tumor markers are thus analyzed routinely to make a presumptive diagnosis. These coupled with extensive gross sampling of the tumor specimens, and a diligent histopathological examination may aid in an accurate diagnosis of a malignant neoplasm arising in MCTs.

Mature cystic teratomas (MCTs) are the most common benign germ cell tumors of the ovary in women of reproductive age. These comprise 10–20% of ovarian neoplasms globally (1). Malignant transformation is very rare, occurring in only 0.17–1.4% of the cases (2). Majority of these reported cases are carcinomas, predominantly squamous cell carcinoma. However, carcinoid tumors, malignant melanomas, sarcomas of various types, as well as carcino-sarcomas have also been reported to arise in MCTs. High grade lymphomas are among the least common transformations. Only six cases of diffuse large B -Cell lymphoma (DLBCL) arising in a mature ovarian cystic teratoma have been reported since 1986 (2).

Preoperative detection of malignant transfor-mation of MCTs is difficult. This increases the likelihood of tumor spread, with poor response to available therapeutic modalities, and decreasing patient survival rates (3). Therefore, it is imperative to make an accurate histopathological diagnosis of a malignant transformation occurring in an MCT as early as possible.

Herein, we describe a case of a high grade B cell non- Hodgkin’s lymphoma arising in a mature ovarian cystic teratoma.

## Case presentation

A 45 -years -old Asian female, presented to her local physician with a history of generalized abdominal pain, and abdominal fullness. An abdomino-pelvic ultrasound demonstrated a pelvic mass measuring 8.2 x 6.7 x 5.2 cm, right to the midline, likely ovarian in origin. A subsequent abdominal CT scan revealed a right iliac fossa mass suggestive of a large hemorrhagic ovarian cyst or a dermoid cyst.

**Fig. 1:**
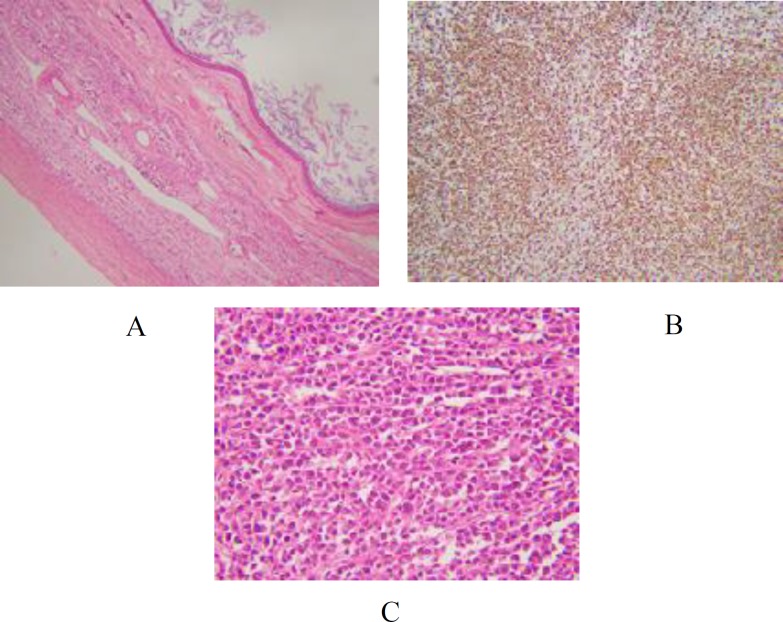
H &E stained sections from the cyst and the nodule. A: Cyst wall showing stratified squamous epithelium and keratin flakes; B: Low power view showing sheets of round blue cells (10x); C: High power view showing tumor cells with hyperchromatic nuclei and scant cytoplasm (40x

Given the size of the ovarian mass, a right sided salpingo-oophorectomy was planned, and performed. Gross pathological examination revealed a unilocular cyst measuring 12.5 x 10.5 x 8.0 cm, with a smooth and intact capsule. The cyst lumen was filled with sebaceous material and hair. Except for a 5.0 cm Rokitansky nodule, no other nodule or papillary structures were identified. Microscopic examination of the formalin fixed, paraffin-embedded multiple sections of the right ovarian mass revealed an admixture of haphazardly arranged mature tissues originating from different germ layers. There were streaks and islands of dermal tissue with small pools of keratin, and the presence of skin adnexal structures (Figure 1A). Ovarian stroma identified was in the form of bands and sheets. Sections from the Rokitansky nodule also demonstrated the presence of incidental foci that displayed a monomorphic population of round blue cells. The tumor cells were arranged in nests and sheets, were large sized and had scant, eosinophilic cytoplasm, pleomorphic, hyperch-romatic nuclei with frequent atypical mitoses (Figures 1 B and C).

**Fig. 2:**
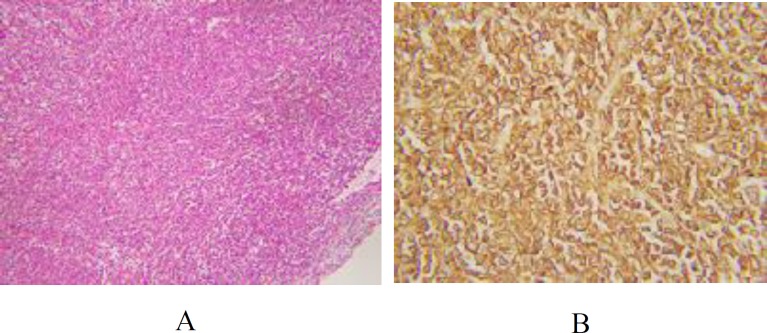
Immunohistochemical stains. A: Ki-67 highlighting more than 90% of tumor cells; B: CD-20 positive in tumor cells, confirming B-cell origin

The adjacent ovarian parenchyma and fallopian tube also showed infiltration by nests of similar round blue cells. There was evidence of lymphovascular invasion. Based on morphological features, a differential diagnosis of germ cell tumor, neuroendocrine tumor, and lymphoma was raised. A panel of immunohistochemical markers was applied. Leukocyte common antigen (LCA), and CD-20 were diffusely positive in the round blue cells (Figure 2A), confirming their B lymphoid series cell origin. Ki-67 proliferation index amounted to 90%, highlighting the brisk mitotic activity in the lymphoid cells (Figure 2B). PLAP, CD-117, CD-30, and synaptophysin immunohis-tochemical stains were negative ruling out the possibility of germ cell tumors and neuroendocrine neoplasms, respectively.

Based on histomorphological and immunoh-istochemical features, a diagnosis of MCT with high grade B-cell non Hodgkin’s lymphoma was rendered.

## Discussion

MCT is not a rare occurrence, it accounts for about 20% of ovarian tumors. MCTs are believed to arise from germ cells by failure of meiosis II or from a premeiotic cell in which meiosis I has failed. These are composed of well-differentiated derivations from at least two of the three germ cell layers (i.e., ectoderm, mesoderm, and endoderm). They contain developmentally mature skin complete with the pilosebaceous apparatus. Other elements that may be seen are most often blood, fat, bone, nails, teeth, cartilage, neural tissue, thyroid tissue, respiratory and intestinal type of epithelium (4). While most patients with MCT show no signs or symptoms, abdominal discomfort, a palpable mass, and pain can frequently occur. MCTs are usually benign, but can undergo malignant transformation in <2% of cases.

The most common malignancy arising in MCTs is squamous cell carcinoma, which consists of about 75% of all malignant transformations (5). This is followed by a large spectrum of adenocarcinomas (6). However, malignant transformation to high grade lymphomas is rarely if ever encountered. Only six cases of high grade B cell non-Hodgkin’s lyphoma arising in MCTs have been reported to date (2).

High grade B cell lymphomas previously reported have mostly been of primary ovarian origin. The present cases, and the only six cases previously reported however, are of a high grade B cell non- Hodgkin’s lymphoma arising in an ovarian MCT.

Previous studies have identified some risk factors for malignant transformations in MCTs; these factors broadly include older age at presentation, post-menopausal status, and raised serum tumor markers; especially serum CA-125 levels (7). Other findings consistently seen in patients with malignantly transformed MCTs include larger tumor masses with grossly visible areas of hemorrhage and necrosis.

Preoperative diagnosis of MCT of the ovary can be made through radiologic findings considering the frequent presence of the adipose tissue, hair, bone, and cartilage within the benign tumor. However, MCTs with malignant transformations are not easily recognized. In most cases, a definitive diagnosis is possible only on postoperative histopathological examination of multiple tissue sections. Similarly, the presence of incidental foci of a high grade B-cell non-Hodgkin’s lymphoma in the present case suggests that in teratomas, sufficient sampling from solid components is important to not miss the microscopic small areas showing evidence of a malignant transformation.

The optimal treatment strategy for such cases remains the main challenge. Surgical excision is regarded as the first approach (8). Based on the literature, prognosis of MCTs with malignant transformations is poor (9). Our review of recent reported cases has shown different approaches including a combination of surgery, chemotherapy, and/ or immunotherapy. Patient outcome is unpredictable even when favorable treatments are used.

In conclusion, a high grade B-cell non-Hodgkin’s lymphoma arising in an ovarian MCT is extremely rare as demonstrated in the present case. Moreover, preoperative identification of MCTs with malignant transformations is not easy. Patient response to the available therapeutic modalities in such cases is poor, and so are the patient survival rates. Therefore, meticulous examination of multiple tissue sections followed by a prompt and accurate histopathological diagnosis could prevent early tumor spread, and may result in a better disease course for the patient.

## Conflict of Interest

The authors declared no conflict of interest.
